# Inflammatory Network Remodeling in AML Is Driven by Neutrophil-Centric Self-Amplifying Circuits

**DOI:** 10.3390/ijms27198703

**Published:** 2026-09-29

**Authors:** Shuqing Wang, Kaini Shen

**Affiliations:** Department of Hematology, Peking Union Medical College Hospital, Chinese Academy of Medical Sciences and Peking Union Medical College, Beijing 100730, China

**Keywords:** acute myeloid leukemia, inflammatory microenvironment reprogramming, prognosis, inflammatory risk score

## Abstract

Acute myeloid leukemia (AML) progression involves remodeling of the bone marrow inflammatory microenvironment, yet prior studies have focused on individual cytokines rather than coordinated network-level communication. This study aimed to systematically map inflammatory network reprogramming in AML and develop a network-derived prognostic tool. Single-cell RNA sequencing was performed on bone marrow samples from three newly diagnosed AML patients and six healthy donors (47,525 cells); ligand–receptor communication was then analyzed using CellChat. Communication dominance shifted from T-cells to myeloid populations, with networks restructured into neutrophil-centric computationally predicted self-amplifying circuits, exemplified by CXCL8–CXCR2 and CCL3–CCR1 axes. Despite the limited sample size of the scRNA-seq cohort (3 AML patients and 6 healthy donors), which may limit the generalizability of the detailed cellular and network findings, the subsequent protein-level Luminex validation in a larger cohort of 46 patients and 26 healthy controls provides robust support for the key findings. An inflammatory risk score (IRS) was constructed using LASSO-penalized Cox regression and evaluated for prognostic performance and integration with the European LeukemiaNet 2022 (ELN2022) classification. Luminex profiling confirmed dysregulation of key mediators, including reduced CCL3, CCL4, CXCL8, and IL-1A together with elevated CXCL10 in AML, and revealed substantial de-modularization of the cytokine correlation network. The IRS, based on CXCL8, CCL2, and IL-1A, achieved a concordance index of 0.729 for overall survival and stratified the ELN2022 intermediate-risk category into distinct prognostic subgroups. Inflammatory network pattern analysis provides a complementary dimension to genetic risk stratification in AML. The IRS captures microenvironment-derived prognostic information not reflected by current classification systems, though prospective multicenter validation is essential before clinical application.

## 1. Introduction

Acute myeloid leukemia (AML) is a heterogeneous hematological malignancy with poor long-term survival despite intensive multimodal therapy [1,2]. Beyond the genetic heterogeneity of malignant cells, the bone marrow microenvironment—particularly inflammatory networks involving immune cells—actively shapes disease progression, therapeutic resistance, and relapse [3,4]. Dysregulated inflammatory signaling orchestrates multiple pathological processes, including leukemic blast survival, recruitment of immunosuppressive populations, T-cell dysfunction, and disruption of normal hematopoiesis [5,6,7]. Individual mediators such as interleukin IL-1A, IL-6, IL-8, and C-C motif ligands (CCL) 2 and 3 have been linked to adverse prognosis. However, understanding of how these factors operate as coordinated networks rather than isolated signals remains incomplete, hindering mechanism-based prognostic tools and therapies.

Traditional approaches have focused on individual cytokines, identifying IL-6, IL-8, CCL2, and CCL3 as adverse markers [8,9]. While clinically relevant, these studies overlook a fundamental principle: inflammatory responses function as integrated networks, not independent signals [10,11]. In normal physiology, cytokines operate through coordinated circuits involving multiple cell types, ligand–receptor pairs, and feedback loops. Individual molecules exhibit redundancy and context-dependency; the same cytokine can promote opposing effects depending on cellular composition and concurrent signals [12,13]. Thus, single-marker approaches, while identifying prognostically relevant factors, may miss critical organizational patterns that better capture disease biology. Emerging evidence from solid tumors suggests that intercellular communication networks predict therapeutic responses better than isolated cell abundance or cytokine levels [14,15]. Whether AML exhibits analogous network-level remodeling and whether such patterns hold prognostic value beyond individual biomarkers remain unexplored.

Deciphering inflammatory network organization in AML has multiple translational implications. First, understanding rewired cellular crosstalk may provide mechanistic insights into progression and resistance, identifying rate-limiting nodes, feedback loops, and cell-type-specific vulnerabilities [16,17]. Second, if dysregulation operates as coordinated network patterns, risk stratification based on multifactor signatures may outperform single-marker approaches, particularly for patients with ambiguous prognosis under current systems [18,19]. Third, characterizing critical hubs could prioritize immunomodulatory targets beyond individual cytokine blockade toward dismantling key communication axes. However, systematic characterization requires integration of cellular-resolution profiling with quantitative protein-level validation in clinically annotated cohorts—an approach not yet comprehensively undertaken.

Despite advances in genetic risk stratification, whether the systemic remodeling of the AML inflammatory network can be captured at the protein level and translated into a clinically applicable prognostic tool remains largely unexplored. We therefore hypothesized that AML reshapes the bone marrow inflammatory microenvironment from a modularly organized cytokine network into a myeloid-skewed, self-amplifying pro-inflammatory axis, and that the intensity of this remodeling carries prognostic information independent of European LeukemiaNet 2022 (ELN2022) genetic risk stratification [20]. To test this hypothesis, we integrated single-cell transcriptomic profiling with multiplex cytokine quantification of bone marrow supernatant to derive and internally validate a parsimonious inflammatory risk score. First, we performed single-cell RNA sequencing on bone marrow samples from three newly diagnosed AML patients and six healthy donors to map cellular composition and ligand–receptor communication. We then validated these findings at the protein level using Luminex multiplex assays in an expanded cohort of 46 AML patients and 26 healthy controls, by measuring the functional secretory changes associated with the inferred microenvironmental remodeling. Finally, we constructed an inflammatory risk score (IRS) to evaluate its prognostic performance and its potential to refine the ELN2022 risk classification. We anticipate that this integrative framework will provide novel insights into the AML microenvironment and offer a clinically applicable tool to improve risk stratification.

## 2. Results

### 2.1. Patients Cohort

The study cohort comprised 46 newly diagnosed AML patients and 26 healthy controls. Median age was 51 years (range, 14–86), with 20 (43.5%) males. Median diagnostic counts were as follows: white blood cell (WBC) count, 12.1 × 10^9^/L (1.3–163.9); hemoglobin, 86.5 g/L (32–142); platelets, 36 × 10^9^/L (7–274); and bone marrow blasts, 45.2% (15.5–97.0). According to the ELN2022 classification, 23 (50.0%) were favorable-risk, 13 (28.3%) intermediate, and 10 (21.7%) adverse. By French–American–British (FAB) criteria, subtypes were M1 (*n* = 3, 6.5%), M2 (*n* = 32, 69.6%), M4 (*n* = 5, 10.9%) and M5 (*n* = 6, 13.0%). Molecular/cytogenetic profiling (*n* = 43; the remaining 3 patients declined testing owing to financial constraints) identified frequent alterations including RUNX1::RUNX1T1 (*n* = 14), CBFB::MYH11 (*n* = 2), NPM1 (*n* = 13), CEBPA (*n* = 7), FLT3 (*n* = 9), KIT (*n* = 5), IDH1/2 (*n* = 5), ASXL1 (*n* = 3), DNMT3A (*n* = 3), TET2 (*n* = 3), EZH2 (*n* = 2), NRAS (*n* = 2), and RUNX1 (*n* = 2). Most patients received intensive anthracycline/cytarabine-based induction and consolidation; a subset received hypomethylating agents plus venetoclax. Among 44 response-evaluable patients, 38 achieved complete remission (CR), CR with incomplete recovery, or CR with measurable residual disease (MRD) negativity, yielding a composite CR rate of 86.4% (*n* = 44). After a median follow-up of 33.5 months (reverse Kaplan–Meier) in 45 patients, 12 (26.7%) died (*n* = 45), with mortality increasing by ELN2022 risk (favorable 3/23 [13.0%]; intermediate 3/13 [23.1%]; adverse 6/10 [60.0%]) (Supplementary Table S1 and Figure S1). The 26 healthy controls had median age 56.5 years (23–77) and were 50% male, with no significant differences vs. patients in age (*p* = 0.08) or sex (*p* = 0.77).

### 2.2. Single-Cell Profiling Reveals Myeloid Skewing and T-Cell Depletion in AML

**Figure 1:**
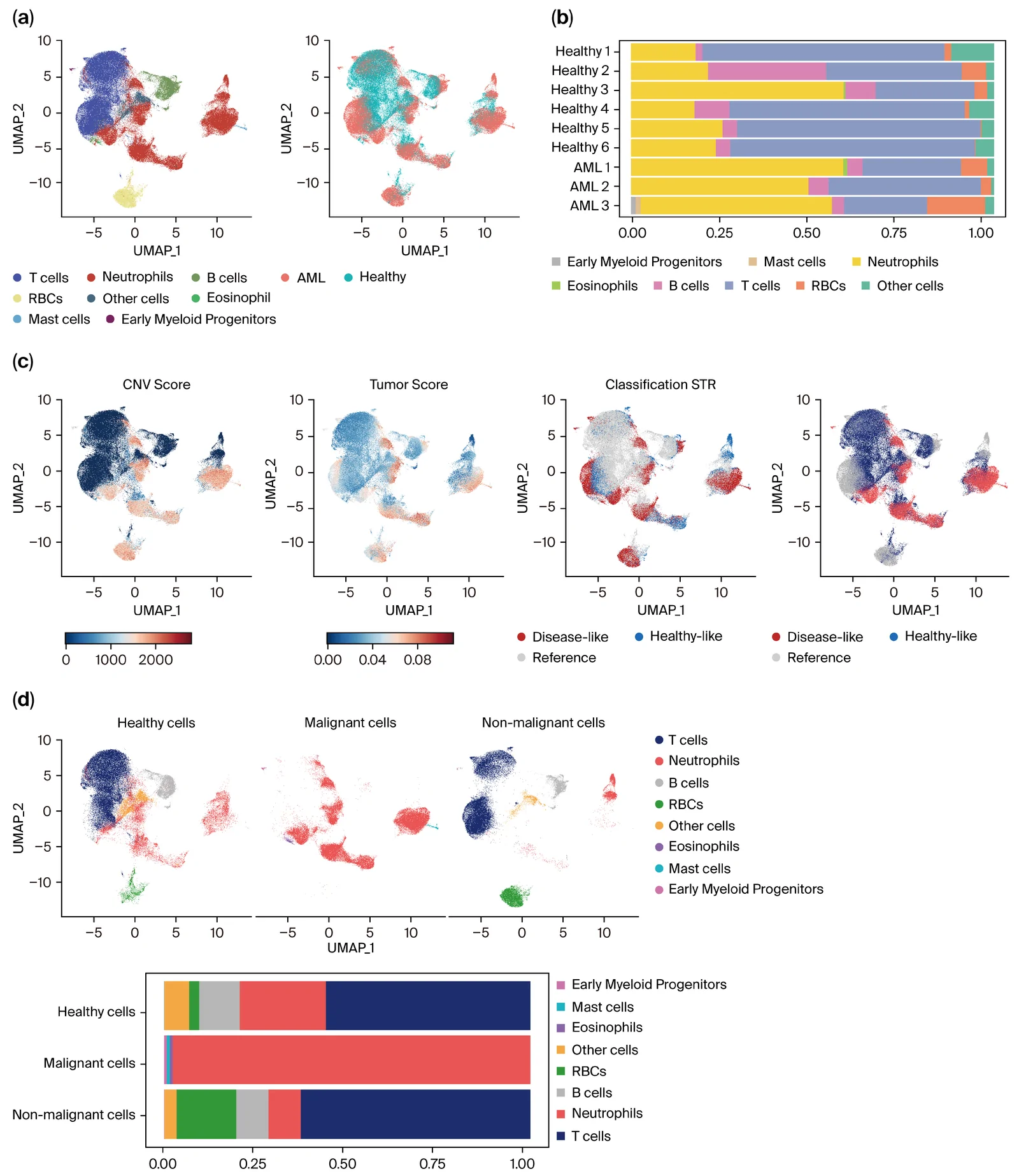
Single-cell transcriptomic landscape and malignant architecture of the AML bone marrow microenvironment. (**a**) Uniform manifold approximation and projection (UMAP) plots of 47,525 bone marrow cells, color-coded by eight major hematopoietic lineages (left) and sample groups (right; AML vs. healthy). (**b**) Stacked bar chart illustrating the relative proportions of hematopoietic cell types across individual healthy donors (*n* = 6) and treatment-naïve AML patients (*n* = 3), capturing a distinct myeloid-lymphoid imbalance. (**c**) Computational identification of malignant clones integrating inferred copy number variation (CNV) scores (left), tumor signature module scores (Tumor Score) (middle), and transcriptional classification models (Classification STR) (right), yielding a final consensus cell classification defining disease-like, healthy-like, and reference cell states. (**d**) UMAPs (top) and quantified compositional bars (bottom) partitioning the cellular atlas into healthy, malignant, and non-malignant compartments.

Single-cell RNA sequencing (scRNA-seq) of 47,525 bone marrow cells from three patients with AML and six healthy donors resolved eight major hematopoietic populations (Figure 1a). Cell type annotation was performed based on the expression of lineage-specific marker genes, as detailed in Supplementary Figure S2. Transcriptional profiling distinguished AML-derived from healthy cells, confirming disease-associated remodeling. Compositional analysis revealed a striking myeloid–lymphoid imbalance: AML marrow showed marked neutrophil expansion and T-cell depletion versus controls, suggesting a niche favoring myelopoiesis at the expense of adaptive immunity (Figure 1b). To accurately distinguish malignant cells from their non-malignant counterparts, three computational approaches were integrated: inferred copy number variation (CNV) scores, tumor signature module scores, and a co-embedding-based transcriptional classification method designated “STR” (Single-cell Transcriptomic Reference-based classification). Cumulative CNV scores for individual cells were calculated using the inferCNV R package, serving as a quantitative indicator of genomic variation at the single-cell level (Figure 1c, left). Tumor signature scores were computed using the AddModuleScore function from Seurat, based on a gene set significantly upregulated in TCGA LAML tumors compared to normal whole blood (Figure 1c, middle). Finally, cell states were defined via the “STR” classification using co-embedding clustering: cells were classified as disease-like (malignant) if they belonged to a cluster containing >80% of cells from an AML sample, and healthy-like (non-malignant) if they co-clustered with >80% of cells from healthy donors. The remaining cells were classified as “Reference” cells (Figure 1c, right). The final malignant versus non-malignant designation of each cell was assigned by the STR classification, with the CNV and tumor signature scores serving as independent, orthogonal corroboration: disease-like clusters consistently exhibited elevated cumulative CNV burden and tumor signature scores relative to healthy-like clusters. By cross-referencing the three approaches, the early myeloid progenitor compartment was almost entirely malignant, whereas T cells—though reduced-remained predominantly non-malignant and healthy (Figure 1d), indicating that AML-driven remodeling operates via both malignant clone expansion and active suppression of benign lymphopoiesis.

### 2.3. AML Reprograms Neutrophil and T-Cell Subpopulations Toward a Neutrophil-Dominated Inflammatory Signaling Network

**Figure 2:**
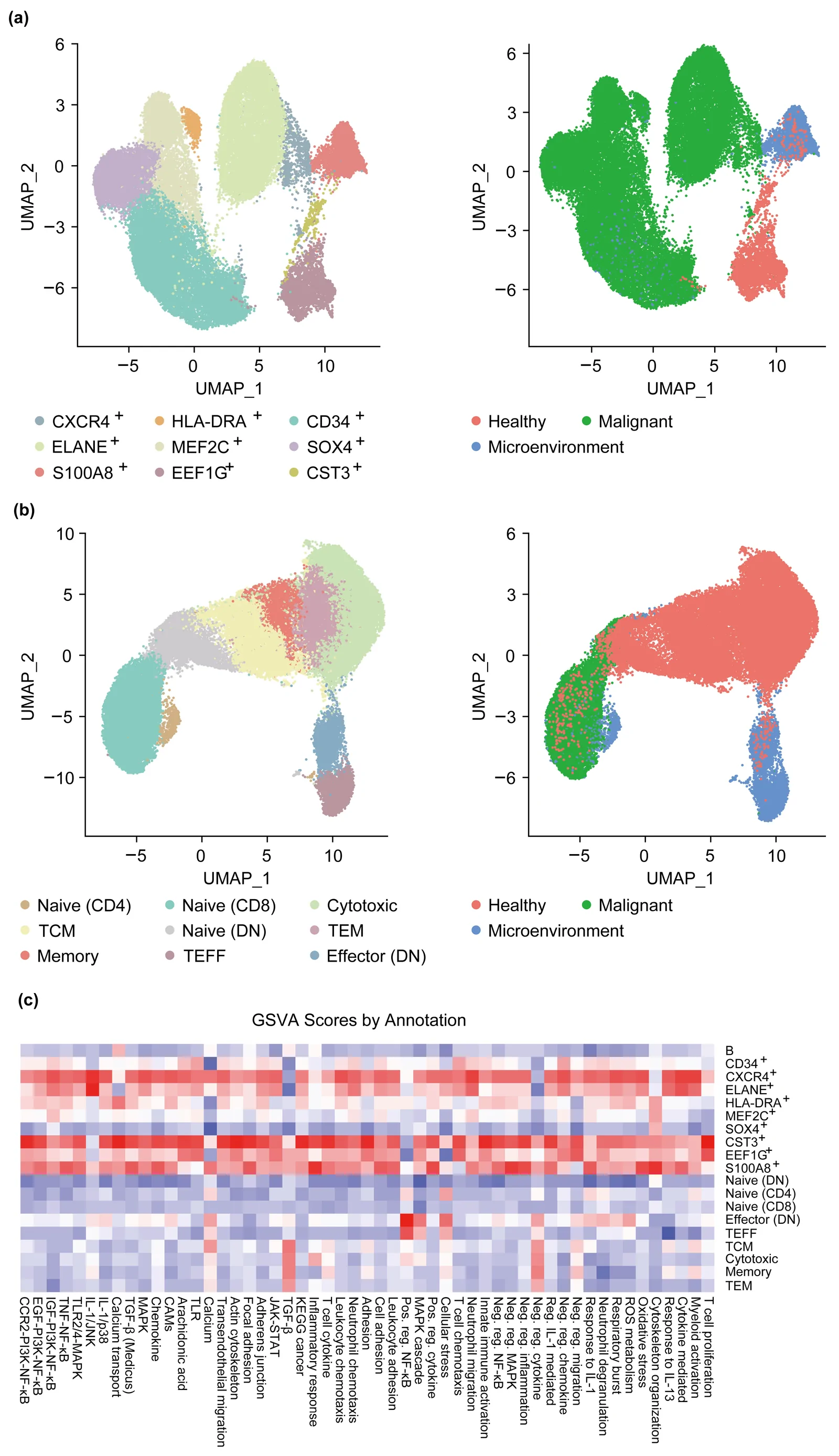
Single-cell dissection of neutrophil and T-cell landscape remodeling and functional reprogramming in the AML marrow. (**a**) UMAP visualization of the neutrophil compartment resolving nine distinct phenotypic subsets (left), including six immature (CD34+, CXCR4+, HLA-DRA+, ELANE+, MEF2C+, and SOX4+) and three mature (EEF1G+, S100A8+, and CST3+) clusters, with projections color-coded by sample origin (right; healthy, malignant, and microenvironment). (**b**) UMAP visualization of the T-cell compartment resolving nine distinct phenotypic subsets (left), including naive (CD4, CD8, and DN), memory (TCM, TEM, and Memory), cytotoxic, and effector (TEFF and Effector DN) clusters, with projections color-coded by sample origin (right; healthy, malignant, and microenvironment). (**c**) Heatmap displaying gene set variation analysis (GSVA) scores across the identified neutrophil, T-cell, and B-cell sub-clusters, illustrating distinct activation patterns in inflammatory cascades, degranulation signaling, and homeostatic pathways. The color scale represents GSVA scores, with warmer colors (red) indicating higher GSVA scores and cooler colors (blue) indicating lower GSVA scores, as shown by the color bar.

To dissect ecosystem remodeling in AML, we profiled bone marrow single cells from three patients and six healthy donors using scRNA-seq. Unsupervised clustering of the neutrophil compartment resolved nine distinct subsets: six immature (CD34+, CXCR4+, HLA-DRA+, ELANE+, MEF2C+, and SOX4+) and three mature (EEF1G+, S100A8+, and CST3+) cohorts (Figure 2a). AML marrows exhibited a profound compositional shift away from homeostatic mature subsets (e.g., EEF1G+ neutrophils) toward the marked expansion of immature precursors and activated mature subsets such as S100A8+ neutrophils. Notably, although the EEF1G+, S100A8+, and CST3+ clusters all lacked early progenitor markers (CD34-), they exhibited highly divergent functional trajectories. EEF1G+ neutrophils predominantly expressed transcripts involved in protein biosynthesis and cellular homeostasis, whereas S100A8+ neutrophils significantly upregulated alarmins and pro-inflammatory mediator genes, indicating a distinct shift from a homeostatic to a highly activated functional state induced by the AML microenvironment. Concurrently, the T-cell landscape in AML marrows demonstrated a severe contraction of naïve and functional memory/effector compartments that were otherwise abundant in healthy donors (Figure 2b). Specifically, key antitumoral populations, including the naïve (CD8), cytotoxic, and central memory T (TCM) subsets, experienced marked depletion, indicating a broad collapse of adaptive anti-tumor immunity within the leukemic niche. Gene set variation analysis (GSVA) further linked these compositional shifts to profound functional reprogramming across the microenvironment (Figure 2c). AML-enriched immature neutrophil subsets displayed a constitutively activated, pro-inflammatory profile, characterized by the robust upregulation of neutrophil degranulation and NF-κB signaling cascades (e.g., NF-κB and TNF-NF-κB), alongside the concomitant suppression of baseline homeostatic pathways. In stark contrast, residual T-cell subsets showed a broad downregulation of cellular migration pathways (e.g., actin cytoskeleton and transendothelial migration) and effector functions (e.g., cytokine-mediated and T-cell proliferation), strongly suggesting functional exhaustion or active immune suppression mediated by the dominant myeloid inflammatory network.

### 2.4. Rewired Ligand–Receptor Circuits Drive Systemic Inflammatory Network Remodeling in AML

**Figure 3:**
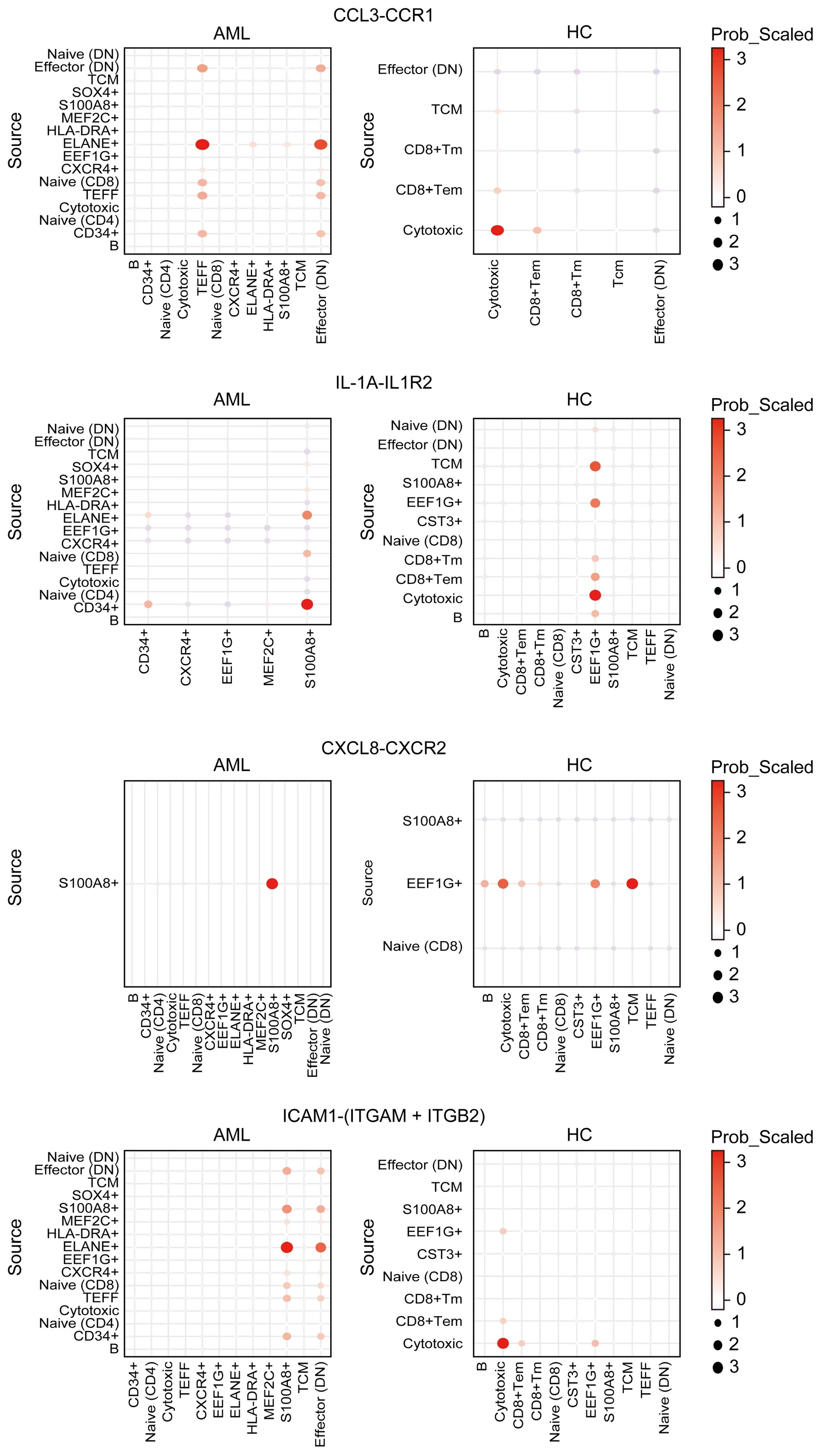
Dot plots showing the inferred intercellular communication probability (color intensity, Prob_Scaled) and interaction strength (dot size) for four signaling axes in AML (left) and healthy controls (HC; right). (CCL3–CCR1) In HC, the network is predominantly driven by lymphoid populations. In AML, the signaling shifts towards an immature myeloid-dominated hub. (IL-1A–IL1R2) In HC, signaling is distributed among EEF1G+ neutrophils and T-cell subsets. In AML, communication is restricted to a dominant paracrine circuit driven by CD34+ early myeloid progenitors targeting S100A8+ neutrophils. (CXCL8–CXCR2) In HC, signaling is mediated by EEF1G+ neutrophils. In AML, communication is restricted to an autocrine loop centered exclusively on S100A8+ neutrophils. (ICAM1–(ITGAM + ITGB2)) The network undergoes a complete transition from a lymphoid-inclusive architecture in HC to a myeloid-dominated interaction in AML.

Single-cell profiling revealed marked alterations in the cellular composition of AML bone marrow, particularly significant changes in T cells and neutrophils. To further investigate the functional interactions among inflammatory and chemokine mediators in AML, we focused on these two cell populations and inferred the ligand-receptor communication networks using CellChat. By comparing AML and healthy groups, we prioritized for detailed analysis those signaling axes that were significantly changed in AML and ranked among the top differential interactions by communication probability. This yielded four axes—CCL3–CCR1, IL-1A–IL1R2, CXCL8–CXCR2, and ICAM1–(ITGAM + ITGB2)—spanning chemokine, interleukin, and adhesion signaling. We then mapped the ligand–receptor topologies of these four pivotal axes to dissect the cellular mechanics driving microenvironmental dysregulation (Figure 3). This intercellular analysis revealed a profound communication rewiring that directly underpins the macro-level systemic network disruption. In healthy bone marrow, communication was highly modular and lymphoid-inclusive. The CCL3 and ICAM1 networks were dominated by functional cytotoxic T-cells, consistent with their homeostatic immune-surveillance role. Meanwhile, CXCL8 and IL-1A signaling pathways were primarily moderated by homeostatic EEF1G+ neutrophils acting as central sending hubs to maintain microenvironmental equilibrium. In the AML microenvironment, these homeostatic architectures collapsed as signaling dominance shifted decisively toward expanded myeloid populations. In the CCL3–CCR1 and ICAM1 networks, immature myeloid progenitors replaced functional T-cells as primary senders; specifically, ELANE+ immature myeloid cells emerged as the dominant sending hub in both axes, with MEF2C+ progenitors co-dominating the ICAM1 adhesive interactome. Concurrently, healthy multicell-moderated pathways contracted into restricted, disease-associated circuits. The CXCL8 pathway degenerated into a putative pathological, autocrine self-amplifying loop restricted entirely within the pro-inflammatory S100A8+ neutrophil subset. In parallel, the IL-1A pathway rewired into a unidirectional paracrine axis, wherein malignant CD34+ early myeloid progenitors acted as the predominant source, heavily targeting S100A8+ neutrophils. Ultimately, these single-cell dynamics demonstrate that AML fundamentally re-routes the cellular conduits of immune communication. By converting lymphoid-moderated networks into myeloid-centric, autonomous, or aberrant paracrine circuits, these abnormal cellular interactions are computationally associated with the global, de-modularized systemic cytokine remodeling that characterizes the leukemic bone marrow.

### 2.5. Multiplex Cytokine Profiling Reveals Prognostic Inflammatory Alterations and Network De-Modularization in AML

**Figure 4:**
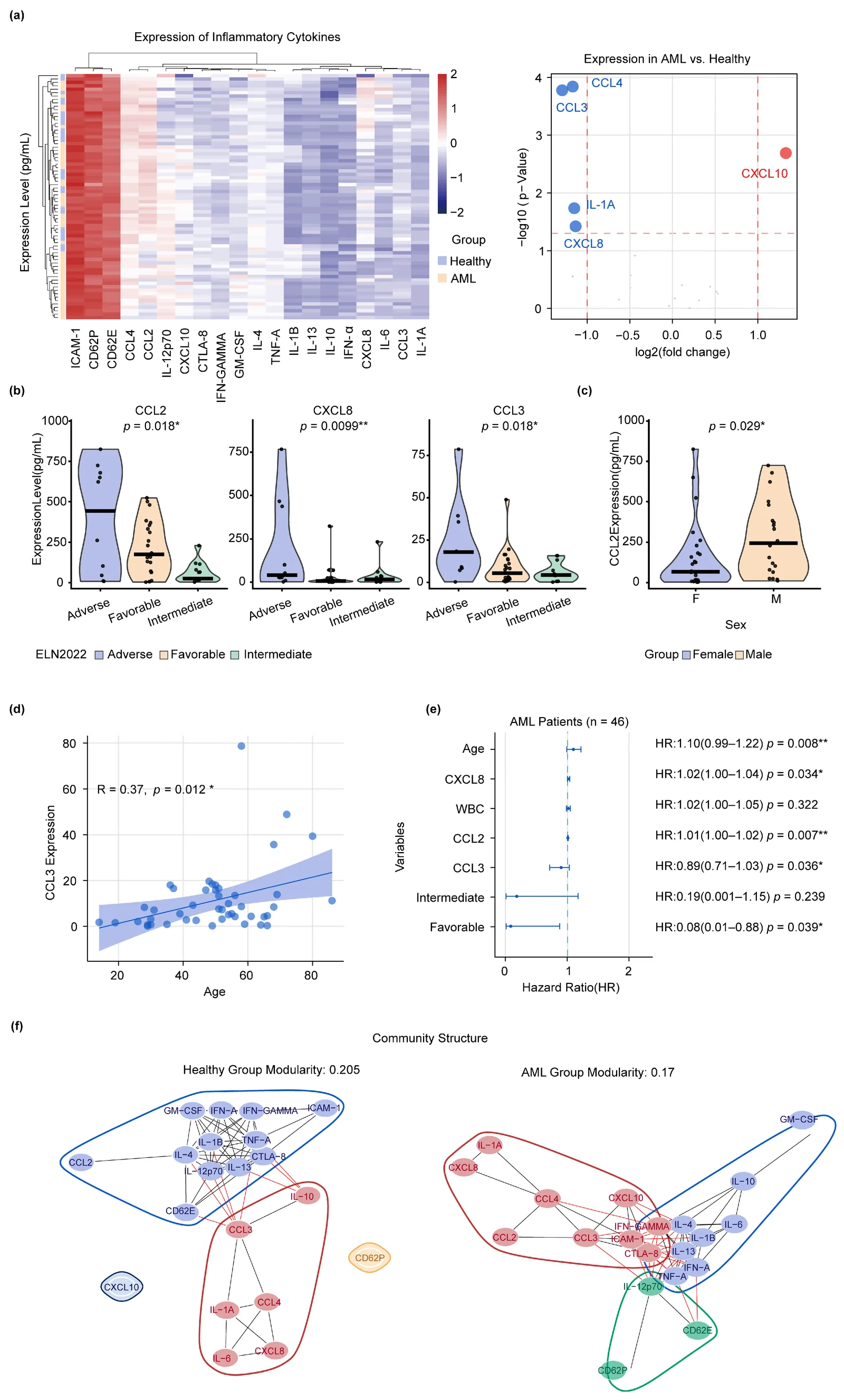
Multiplex Cytokine Profiling Reveals Prognostic Inflammatory Alterations and Network De-modularization in AML. (**a**) Multiplex Luminex profiling of 20 candidate inflammatory mediators in bone marrow supernatants from treatment-naïve AML patients (*n* = 46) and healthy controls (*n* = 26). Left: Unsupervised hierarchical clustering heatmap showing global expression landscapes. Right: Volcano plot identifying the expression of CCL3, CCL4, CXCL10, CXCL8, IL-1A in AML. (**b**) Expression dynamics of bone marrow CCL2, CXCL8, and CCL3 stratified across ELN2022 genetic risk categories, highlighting non-linear pattern enrichment locked in adverse-risk cohorts. (**c**,**d**) Demographic stratifications illustrating significantly higher CCL2 levels in male patients (**c**) and a significant positive Spearman correlation between baseline CCL3 concentrations and advancing patient age (**d**). (**e**) Multivariate Cox proportional hazards forest plot determining the independent prognostic significance of clinical covariates and significantly altered microenvironmental cytokines for overall survival in the AML validation cohort. (**f**) Correlation network analysis mapping global topological community structures and de-modularization from healthy controls (left; modularity Q = 0.205) to the AML microenvironment (right; modularity Q = 0.17). Nodes represent inflammatory mediators; edges represent significant Spearman correlations (|r| ≥ 0.5, *p* < 0.05). The co-optation of the previously isolated CXCL10 node into the central inflammatory signaling hub is highlighted. * *p* < 0.05, ** *p* < 0.01 indicate statistical significance.

To validate at the protein level the scRNA-seq-inferred microenvironmental remodeling—specifically, by assessing whether the altered cellular composition translates into changes in the functional secretory profile—we performed multiplex Luminex profiling on an expanded validation cohort of 46 AML patients and 26 healthy controls (which included the 3 scRNA-seq patients). Consistent with the scRNA-seq prediction of a myeloid-skewed microenvironment with T-cell depletion, protein-level profiling confirmed significantly reduced T-cell-associated chemokines in AML bone marrow supernatant (CCL3, 26.0 vs. 11.2 pg/mL, healthy vs. AML, *p* = 0.0002; CCL4, 266.3 vs. 149.2 pg/mL, *p* = 0.0001). Among myeloid-associated mediators, CXCL10 was drastically upregulated (21.5 vs. 55.4 pg/mL, *p* = 0.002), whereas CXCL8 (159.5 vs. 65.1 pg/mL, *p* = 0.038) and IL-1A (8.6 vs. 4.5 pg/mL, *p* = 0.018) were significantly reduced (Figure 4a). We next interrogated the correlation between these alterations and clinical risk strata. Intriguingly, CCL2, CXCL8, and CCL3 varied significantly across ELN2022 risk groups, with the highest expression levels consistently locked in adverse-risk patients and non-linear inter-group patterns across risk categories (Figure 4b). Demographic stratification showed that CCL2 expression was significantly higher in male patients (Figure 4c), whereas CCL3 levels displayed a distinct positive correlation with advancing age (R = 0.37, *p* = 0.012) (Figure 4d). To assess global clinical impact, we constructed a comprehensive multivariate Cox proportional hazards model incorporating clinical covariates and significantly altered cytokines. Beyond the expected protective effect of ELN2022 favorable status (HR = 0.08, *p* = 0.039), age (HR = 1.10, *p* = 0.008), CXCL8 (HR = 1.02, *p* = 0.034), CCL2 (HR = 1.01, *p* = 0.007), and CCL3 (HR = 0.89, *p* = 0.036) were identified as robust, independent prognostic determinants for overall survival in this cohort (Figure 4e).

Finally, correlation network analysis demonstrated substantial systemic topological remodeling. In this analysis, a “module” represents a community of cytokines with densely interconnected correlations, reflecting a co-regulated inflammatory program. Healthy controls maintained a highly organized, modular architecture (modularity Q = 0.205), wherein CXCL10 existed as a completely isolated, non-interfering node (Figure 4f). In stark contrast, the AML marrow underwent substantial “de-modularization” (modularity Q decreased to 0.17, a 17% relative reduction accompanied by a collapse from four cytokine communities to three), shifting the network into a densely interconnected pathological topology. In this leukemic state, the previously isolated CXCL10 was heavily co-opted into the central network hub alongside CCL3 and ICAM-1, acquiring significant correlations with IFN-γ, IL-4, and CTLA-8, anchoring a self-sustaining inflammatory architecture that defines the diseased microenvironment (Figure 4f).

### 2.6. Individual Bone Marrow Cytokines as Single-Marker Prognostic and Predictive Indicators in AML

**Figure 5:**
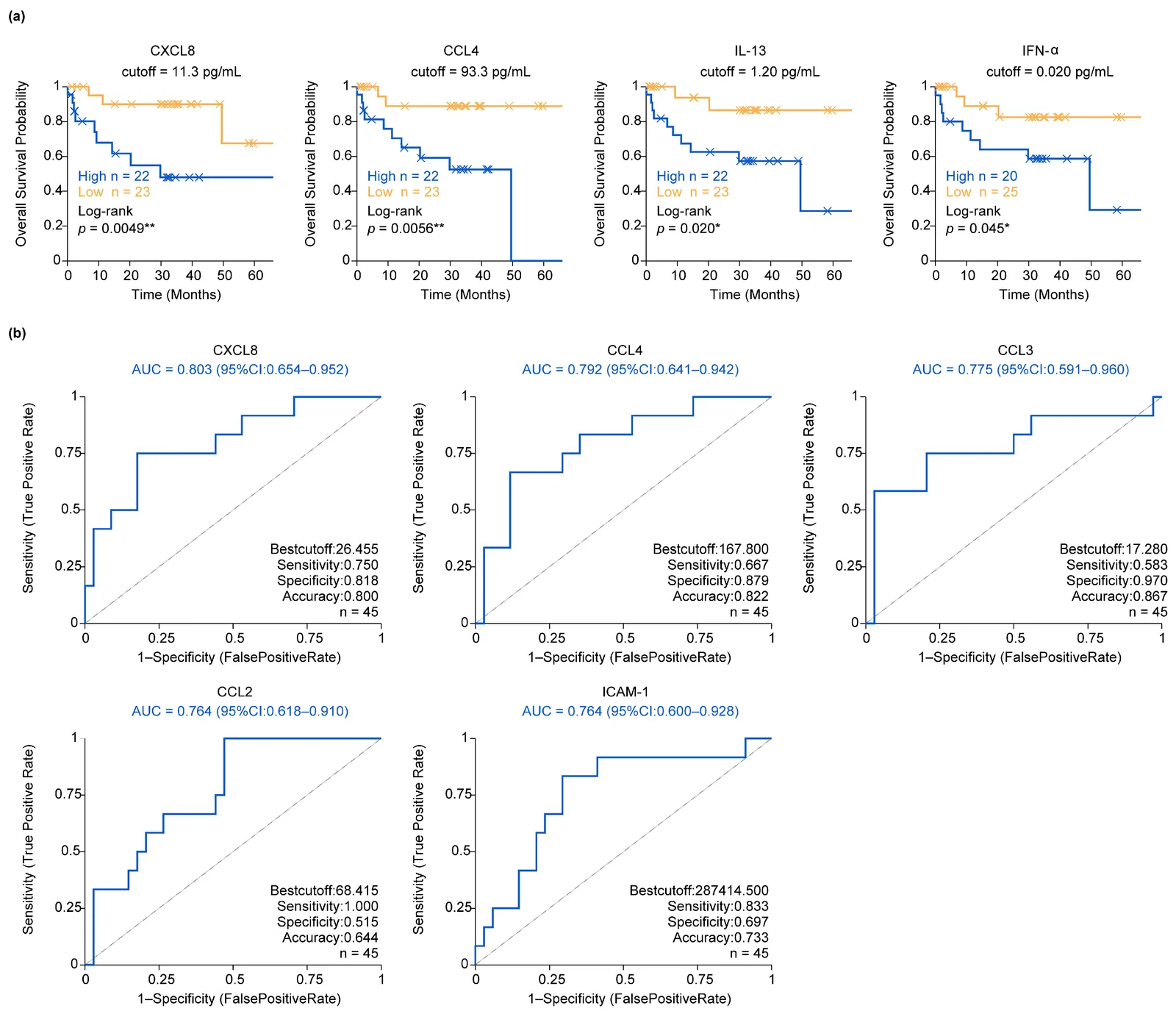
Individual Bone Marrow Cytokines as Single-Marker Prognostic and Predictive Indicators in AML. (**a**) Kaplan–Meier overall survival (OS) curves of AML patients (*n* = 45) stratified by the median bone marrow supernatant concentrations of individual inflammatory mediators. The median concentrations used as cutoff values were 11.3 pg/mL for CXCL8, 93.3 pg/mL for CCL4, 1.20 pg/mL for IL-13, and 0.02 pg/mL for IFN-α. The blue lines represent patients with high expression levels (above the median), and the yellow lines represent patients with low expression levels (below the median). Cross marks indicate censored patients. Statistical significance was determined via the log-rank test. (**b**) Receiver operating characteristic (ROC) curves evaluating the mathematical accuracy and discriminatory performance of individual candidate biomarkers (CXCL8, CCL4, CCL3, CCL2, and ICAM-1) in predicting patient clinical survival status. Each panel is annotated with its respective Area Under the Curve (AUC), 95% confidence interval (CI), optimal cutoff value, sensitivity, specificity, and overall classification accuracy. * *p* < 0.05 and ** *p* < 0.01 denote statistical significance by log-rank criteria.

To identify the most clinically actionable components within the dysregulated bone marrow microenvironment, we systematically screened the prognostic and predictive value of individual inflammatory mediators. Dichotomizing the patient cohort based on individual cytokine expression levels via Kaplan–Meier analysis revealed that several standalone factors could significantly stratify overall survival (Figure 5a). Specifically, CXCL8 (log-rank *p* = 0.0049) and CCL4 (log-rank *p* = 0.0056) served as highly significant indicators of inferior clinical outcomes. Furthermore, aberrant expression profiles of IL-13 (log-rank *p* = 0.02) and IFN-α (log-rank *p* = 0.045) effectively segregated patients into distinct risk trajectories, confirming that individual microenvironmental fluctuations carry profound prognostic signatures. To rigorously evaluate the mathematical accuracy of these individual factors in predicting patient survival status, we performed receiver operating characteristic (ROC) curve analysis (Figure 5b). Multiple single mediators demonstrated meaningful predictive performance, spearheaded by CXCL8 (AUC = 0.803) and CCL4 (AUC = 0.792). Additionally, CCL3 (AUC = 0.775), CCL2 (AUC = 0.764), and ICAM-1 (AUC = 0.764) exhibited robust discriminatory capacity (Figure 5b). Collectively, these findings indicate that individual bone marrow cytokines harbor significant independent prognostic and predictive capacity, highlighting their clinical utility as single-marker indicators in AML.

### 2.7. An Integrated Inflammatory Risk Score Predicts Overall Survival and Refines ELN2022 Risk Stratification in AML

**Figure 6:**
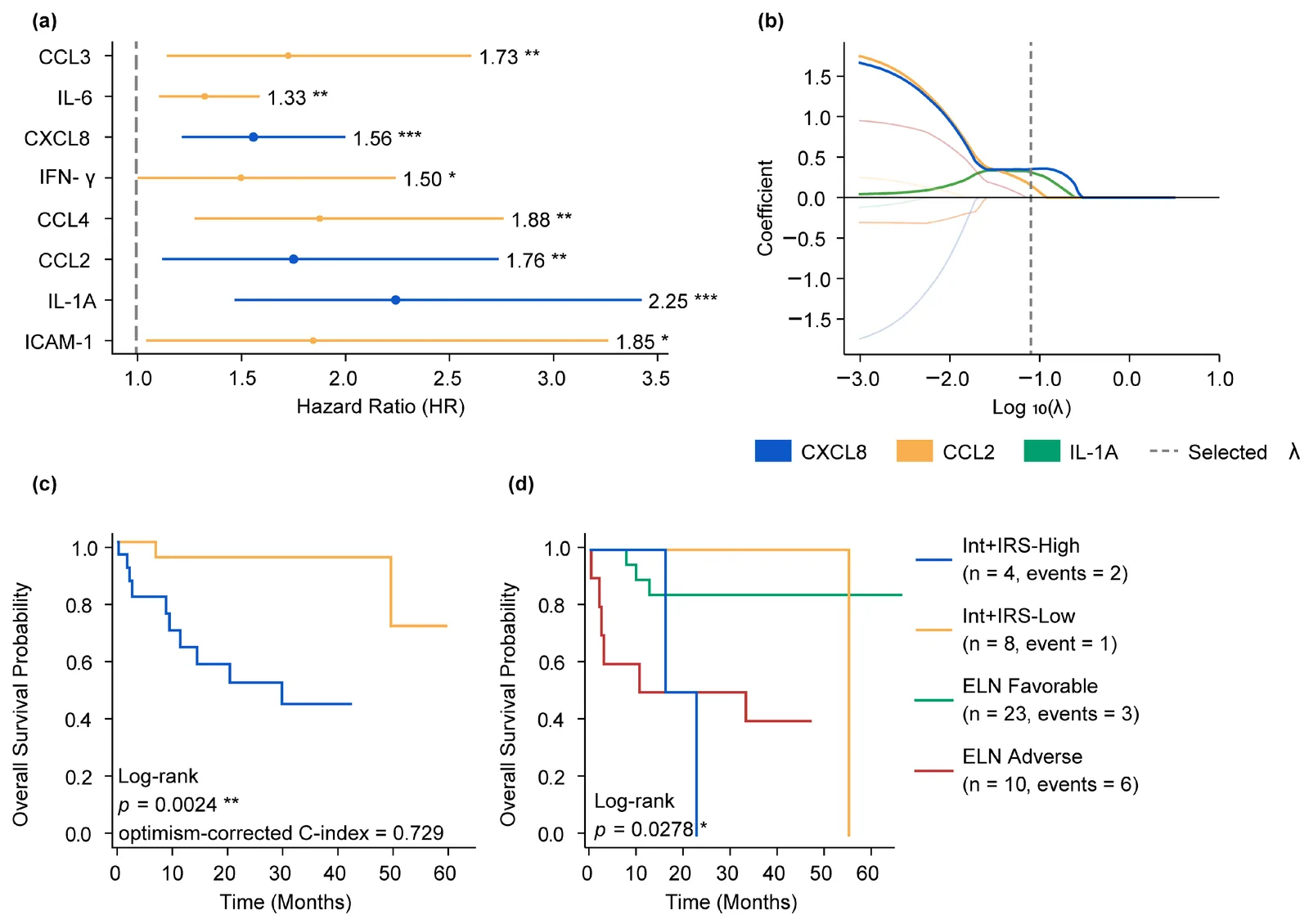
Construction of the Inflammatory Risk Score (IRS) and microenvironment-driven refinement of ELN2022 risk stratification in AML. (**a**) Forest plot illustrating the hazard ratios (HRs) and 95% confidence intervals derived from univariate Cox proportional hazards regression analysis of the eight significantly survival-associated microenvironmental cytokines. (**b**) LASSO-Cox regression analysis with 10-fold cross-validation targeting the candidate prognostic cytokines. The tuning parameter (log_10_(λ)) selection path identifies CXCL8, CCL2, and IL-1A as the optimal, non-redundant variables for risk score formulation. (**c**) Overall survival of the full AML cohort (*n* = 45 with available survival data) dichotomized into IRS-high and IRS-low groups at the cohort median IRS (2.18). The log-rank *p*-value and the optimism-corrected C-index (Harrell’s bootstrap optimism correction, 1000 resamples) are annotated. (**d**) Kaplan–Meier overall survival curves demonstrating the capacity of the microenvironment-derived IRS to refine genomics-based risk categories. * *p* < 0.05, ** *p* < 0.01, and *** *p* < 0.001 denote statistical significance by respective regression or log-rank criteria.

Having established the independent prognostic value of individual cytokines, we next sought to determine whether an integrated multi-marker model could more comprehensively capture the coordinated inflammatory network and improve risk stratification; to this end, we first screened the log_2_(x + 1)-transformed candidate cytokines using univariate Cox proportional hazards regression. Eight bone marrow cytokines were identified as significantly associated with altered overall survival (all Hazard Ratio [HR] > 1), with the most pronounced hazard effects observed IL-1A (HR = 2.25, *p* < 0.001), CCL4 (HR = 1.88, *p* < 0.01), and ICAM-1 (HR = 1.85, *p* < 0.05) (Figure 6a). To eliminate multi-collinearity and avoid overfitting among these significant candidates, we applied least absolute shrinkage and selection operator (LASSO)–Cox regression analysis with 10-fold cross-validation. At the optimal tuning parameter (λ.min), the model retained three non-redundant, highly predictive factors—CXCL8, CCL2, and IL-1A (Figure 6b). All eight candidate cytokines were individually significant in univariate Cox analysis; however, substantial inter-cytokine correlation was observed (Pearson r = 0.33–0.87). Among the retained cytokines, IL-1A and CXCL8 exhibited the strongest univariate effects (HR = 2.25 and 1.56, respectively), whereas CCL2 was retained owing to its relative independence from the other candidates (maximum Pearson r = 0.58; variance inflation factor = 4.2). The five excluded cytokines (CCL4, CCL3, IL-6, ICAM-1, and IFN-γ) were highly correlated with the retained variables (r = 0.60–0.82) and were therefore redundant for prognostic modeling. The resulting formula derived the Inflammatory Risk Score (IRS) as follows:

IRS = 0.1672 × log_2_(CXCL8 + 1) + 0.1600 × log_2_(CCL2 + 1) + 0.3208 × log_2_(IL-1A + 1)

The inflammatory risk score showed good discriminative ability for overall survival, with an optimism-corrected C-index of 0.729. Kaplan–Meier analysis revealed that the IRS effectively dichotomized the cohort, showing a highly significant survival disadvantage for the IRS-high group compared to the IRS-low group, with a median IRS value of 2.18 (log-rank *p* = 0.0024; Figure 6c). Crucially, we evaluated whether this microenvironment-derived score could complement current genomics-based risk classifications by interrogating its performance within the heterogeneous ELN2022 intermediate-risk category. The IRS successfully resolved this ambiguous clinical group into two distinct prognostic strata. Intermediate-risk patients with a low IRS (*n* = 8, event = 1) experienced favorable long-term survival that mirrored the ELN2022 favorable cohort (*n* = 23, events = 3). Conversely, intermediate-risk patients with a high IRS (*n* = 4, events = 2) experienced rapid disease progression and poor survival trajectories overlapping with the ELN2022 adverse cohort (*n* = 10, events = 6) (log-rank *p* = 0.0278; Figure 6d). Together, these data demonstrate that the IRS captures crucial, inflammation-mediated prognostic heterogeneity that is not reflected by autonomous genetic risk systems alone, identifying a subset of nominally intermediate-risk patients who may benefit from early treatment intensification.

## 3. Discussion

To our knowledge, this is the first study to integrate single-cell ligand–receptor topology with protein-level network validation in AML. Consistent with our hypothesis, our findings reveal that AML fundamentally reprograms immune communication through three coordinated transformations: a cellular dominance shift from T-cells to myeloid populations, reconstruction of signal networks into putative self-amplifying pathological circuits (computationally inferred autocrine signaling loops within the myeloid compartment, exemplified by the CXCL8–CXCR2 axis), and functional outputs becoming cell type-dependent rather than molecule-specific. These organizational changes, orthogonally validated at the protein level in an expanded cohort, underscore that inflammatory dysregulation in AML operates as coordinated network patterns rather than isolated cytokine alterations.

The three identified transformations establish a leukemia-permissive microenvironment. The shift from T-cell–centered to myeloid-dominated circuits disrupts immune surveillance and amplifies immunosuppressive signals via expanded immature neutrophil populations [21,22,23,24]. Reconstruction into self-amplifying pathological circuits perpetuates myeloid expansion independent of sustained stimuli [25,26,27], potentially explaining residual immune dysfunction after blast clearance [28,29]. Critically, cell type-dependent rather than molecule-specific functional outputs challenge the traditional view of fixed cytokine activities, emphasizing that network functions emerge from cellular context [12]. Notably, the integrative design also resolves an apparent discrepancy between cellular and bulk views: while single-cell inference identified an activated CXCL8–CXCR2 autocrine circuit within the expanded S100A8+ neutrophil subset in AML, total CXCL8 protein in bone marrow supernatant was reduced, because the dominant CXCL8-sending population in healthy marrow—the homeostatic EEF1G+ neutrophil subset—contracts upon leukemic transformation. Per-cell activation of a signaling circuit therefore does not translate into increased bulk cytokine output when the cellular population dominating its production is lost—a compositional effect that only a network-level, cell-type-resolved design can reveal. Taken together, our network analysis generates the hypothesis that these interconnected transformations may form a putative self-reinforcing inflammatory state, potentially contributing to leukemic survival and impaired antitumor immunity [30,31].

Translating single-cell discoveries into clinical insights requires rigorous validation. While scRNA-seq provides cellular resolution, transcript abundance does not always correlate with protein expression, and our discovery cohort necessitated orthogonal validation in an expanded cohort [32,33]. Our Luminex validation addressed these concerns through orthogonal quantification and larger sample size [34]. This discovery-validation architecture contrasts with many AML single-cell studies confined to transcriptomics without protein-level confirmation [2,35].

The inflammatory risk score (IRS) represents a shift from single-marker to network-based prognostication. Unlike individual mediators [8,9,36], the IRS integrates CXCL8, CCL2, and IL-1A selected by LASSO-Cox regression to capture coordinated network dysregulation [37]. While ELN2022 reflects intrinsic tumor biology, the IRS captures microenvironment-derived risk signals independent of genetics. Its utility includes identifying high-risk patients for treatment intensification and refining ambiguous genetic risk; however, prospective multicenter validation is essential before clinical implementation [38]. A strength of this study is the concordant use of two orthogonal platforms on the same patient cohort: scRNA-seq defined the cellular sources of the dysregulated mediators, and multiplex Luminex profiling confirmed the corresponding directional changes at the protein level in the entire cohort. The IRS was built exclusively on mediators that had been identified at the single-cell level and validated at the protein level, rather than on statistical screening alone.

Beyond prognostication, our characterization suggests rational therapeutic targets. Self-amplifying CXCL8–CXCR2 and CCL3–CCR1 circuits can be disrupted pharmacologically—CXCR2 antagonists [39] and Food and Drug Administration (FDA)-approved CCR1 antagonists may interrupt immunosuppressive myeloid accumulation [40,41]. However, systemic inhibition risks infection in immunocompromised AML patients [42], network redundancy may require combination strategies [43], and integration with existing therapies needs systematic evaluation [44]. Nonetheless, our findings provide a rational framework for designing such studies.

This study has limitations.

First, the scRNA-seq discovery cohort (*n* = 3) limits detection of rare subpopulations, though subsequent Luminex validation (*n* = 46) provides statistical robustness [45]. In addition, the healthy-control scRNA-seq data were obtained from a public repository (10x Genomics, cryopreserved samples), whereas the AML samples were processed in-house (BD Rhapsody, fresh samples); despite Harmony-based integration, residual confounding from differences in laboratories, preservation status, and capture platforms cannot be fully excluded, and the between-group single-cell and cell–cell communication findings should therefore be interpreted with caution. Independent scRNA-seq cohorts and the orthogonal Luminex protein-level validation presented in this study partially mitigate, but do not eliminate, this limitation.

Second, the IRS was developed in a single-center cohort without external validation, requiring multicenter prospective confirmation. We attempted RNA-level validation of the IRS in two independent public cohorts (BeatAML, *n* = 649; TCGA-LAML, *n* = 132). Direct coefficient transfer failed to reproduce the protein-level prognostic performance (*C*-index = 0.50), reflecting fundamental protein-RNA discordance and bulk RNA-seq dilution of cell-type-specific cytokine signals. This underscores that the IRS is a protein-level tool requiring prospective multicenter validation using Luminex or equivalent proteomic platforms. To date, no public dataset simultaneously measures CXCL8, CCL2, and IL-1A at the protein level in the bone marrow microenvironment together with survival outcomes, precluding a complete external protein-level validation of the IRS. Most public AML proteomics cohorts employ mass spectrometry on cell lysates, which predominantly captures intracellular proteins and does not reliably detect secreted cytokines in the extracellular compartment from which the IRS was derived. Nevertheless, independent support for the involvement of CXCL8 in the inflammatory biology of the AML bone marrow comes from an aptamer-based affinity proteomics study (SOMAscan) of bone marrow supernatant, which identified CXCL8 among the most significantly altered proteins in AML (log_2_ fold change = 1.84; FDR = 0.001) and as part of the most prominent cytokine–chemokine signaling network associated with the leukemic bone marrow [46]. We note that the direction of the between-group difference in CXCL8 differs between that study and our cohort, in which CXCL8 concentrations were lower in AML patients than in healthy controls. This discrepancy does not affect the interpretation of the IRS: CXCL8 enters the score as a within-AML prognostic variable—higher concentrations identify patients at higher risk within the AML cohort—rather than through the direction of the AML-versus-healthy difference. The external study therefore supports the biological relevance of CXCL8 in the leukemic bone marrow microenvironment without needing to reproduce its direction of change in our cohort. Prospective multi-center validation of the IRS in a larger cohorts with bone marrow supernatant cytokine profiling and long-term survival follow-up is warranted.

Third, our cross-sectional design precludes assessment of network dynamics during treatment or relapse; longitudinal sampling could reveal whether pathological transformations persist or evolve.

Fourth, our analyses are primarily correlative. The identified circuits (e.g., CXCL8-CXCR2 and CCL3-CCR1) are hypotheses generated from computational network analysis rather than established biological mechanisms. Direct functional validation via in vitro and in vivo experiments (such as co-culture, in vivo models, and ex vivo assays) is required to establish their causal roles in AML pathogenesis.

Finally, ligand–receptor inference from gene expression data warrants spatial transcriptomics or multiplexed immunofluorescence validation to demonstrate physical proximity of interacting cells.

In summary, this study provides the first comprehensive map of inflammatory network remodeling in AML, revealing that immune dysregulation operates through coordinated cellular and molecular transformations rather than isolated cytokine perturbations. The IRS provides a clinically applicable tool that outperforms individual biomarkers in predicting survival, establishing inflammatory network pattern analysis as a novel dimension of AML biology and identifying specific molecular axes as rational therapeutic targets. Prospective IRS validation, longitudinal profiling, functional studies, and spatial transcriptomics emerge as future priorities. Ultimately, understanding AML as a disease of dysregulated immune networks rather than simply malignant expansion may open new therapeutic avenues for restoring effective antitumor immunity.

## 4. Materials and Methods

### 4.1. Patient Cohort and Sample Collection

This study enrolled 46 newly diagnosed, treatment-naïve AML patients and 26 healthy controls. Bone marrow aspirates were obtained at diagnosis; supernatants were separated by centrifugation (2000× *g*, 10 min) and stored at −80 °C. For single-cell RNA sequencing (scRNA-seq), bone marrow from three AML patients was profiled in-house; six healthy donors from public dataset GSM5613791, GSM5613793–GSM5613797 served as controls. The study was approved by the Institutional Review Board of Peking Union Medical College Hospital (No. Y0155); written informed consent was obtained from all participants.

### 4.2. Single-Cell RNA Sequencing and Computational Analysis

Single-cell RNA sequencing and data integration. Libraries for the in-house AML samples were generated using the BD Rhapsody WTA platform (BD Biosciences, San Jose, CA, USA) and sequenced on an Illumina NovaSeq 6000 platform (Illumina, Inc., San Diego, CA, USA) using paired-end 150-bp (PE150) sequencing. Raw sequencing reads were processed using the BD Rhapsody Whole Transcriptome Assay Analysis Pipeline (v1.11), with the Genome Reference Consortium Human Build 38 (GRCh38) as the reference genome. The pipeline included read-quality filtering, read annotation, molecule annotation, putative cell identification, and generation of the single-cell gene expression matrix. The resulting gene-by-cell UMI count matrix was used for downstream analyses. The publicly available healthy control datasets were generated using the 10x Genomics platform (10x Genomics, Inc., Pleasanton, CA, USA). After unified quality control and normalization, batch effects across samples and sequencing platforms were corrected using Harmony (v1.2.3). The Harmony-corrected low-dimensional embeddings were subsequently used for clustering and unsupervised UMAP visualization, without using prior cell-type annotations to guide the dimensionality reduction.

Major hematopoietic populations were annotated according to the coordinated expression of lineage-associated marker genes: T cells were annotated by CD3D and TRAC; neutrophils by ELANE, S100A8, and S100A9; B cells by CD19, MS4A1, and JCHAIN; RBCs (erythroid cells) by HBB and HBA1; eosinophils by IL5RA, and CLC; mast cells by TPSAB1 and TPSB2; and early myeloid progenitors by CD34, CD33, and MPO (Supplementary Figure S2).

Following the initial annotation of the major populations using canonical neutrophil lineage markers (e.g., ELANE, S100A8/A9), the neutrophil population was extracted and subjected to unsupervised sub-clustering. The resulting sub-clusters were annotated based on their top differentially expressed genes (DEGs) and representative cell-state features. Notably, genes such as SOX4 and EEF1G were utilized as representative cluster-associated genes to distinguish specific neutrophil subsets, reflecting distinct cellular states, rather than serving as canonical neutrophil lineage markers. Similarly, T-cell subsets were annotated based on the relative expression patterns of canonical marker genes across unsupervised clusters (e.g., CD4, CD8A, CD8B, CCR7, and CD45RA). Specifically, a cluster was designated as “CD4” or “CD8” based on the relative enrichment or depletion of the corresponding marker compared to other T-cell clusters, rather than exceeding a predefined absolute expression cutoff.

Cell-type composition was quantified by counting the number of cells assigned to each annotated population and summarizing their relative proportions (number of cells per population divided by total cells per sample), which were visualized as a compositional bar plot using ggplot2 (v3.3.6).

### 4.3. Multiplex Luminex Profiling

Bone marrow supernatants from all 46 AML patients and 26 controls were assayed using the Luminex 200 system with a 20-plex human cytokine panel (Thermo Fisher Scientific, Waltham, MA, USA): E-Selectin, GM-CSF, ICAM-1, IFN-α, IFN-γ, IL-1α, IL-1β, IL-4, IL-6, IL-8 (CXCL8), IL-10, IL-12p70, IL-13, IL-17A, IP-10 (CXCL10), MCP-1 (CCL2), MIP-1α (CCL3), MIP-1β (CCL4), P-Selectin, and TNFα. Bone marrow supernatants were collected by centrifugation at 2000× *g* for 10 min, stored at −80 °C, and diluted 2-fold prior to analysis. The kit provides magnetic beads covalently coupled with capture antibodies for the biomarkers. The coupled magnetic beads were incubated with the 2-fold diluted bone marrow supernatant samples at room temperature for 30 min at 600 rpm to allow for sufficient binding. Subsequently, the plates were washed three times with wash buffer to remove unbound proteins. Then, biotinylated detection antibodies were added and incubated at room temperature for 60 min to form a sandwich complex. Next, streptavidin-phycoerythrin (SA-PE) conjugates were introduced to form the final detection complex, which was incubated at 600 rpm for 30 min. After a final washing step, the samples were resuspended in Assay Buffer and loaded onto the instrument for detection.

The raw data acquired from the Luminex 200 system were analyzed. Concentrations were determined by four-parameter logistic (4PL) regression using MILLIPLEX Analyst v5.1 (Merck Millipore). The detected data were processed using Python (v3.10.15), and Matplotlib (v3.9.2) was used for data visualization analysis.

### 4.4. Derivation and Internal Validation of the Inflammatory Risk Score (IRS)

Candidate cytokines were screened by univariate Cox proportional hazards regression on log_2_(x + 1)-transformed concentrations, identifying eight mediators significantly associated with overall survival (all HR > 1, *p* < 0.05). To eliminate multicollinearity and prevent overfitting, LASSO-penalized Cox regression (glmnet, α = 1) was fitted on these eight candidates, which were z-score standardized within the training portion of each cross-validation fold; the penalty parameter λ was tuned by 10-fold cross-validation and set at λ.min, the value minimizing the cross-validated partial-likelihood deviance (the more parsimonious λ.1se criterion was not adopted, as it over-shrinks the model with only 12 events). At the optimal λ, three non-redundant cytokines-CXCL8, CCL2, and IL-1A-were retained, and the coefficients, back-transformed to the log_2_(x + 1) scale, defined the inflammatory risk score (IRS): IRS = 0.1672 × log_2_(CXCL8 + 1) + 0.1600 × log_2_(CCL2 + 1) + 0.3208 × log_2_(IL-1A + 1). Patients were dichotomized into IRS-high and IRS-low groups at the cohort median IRS (2.18). Internal validation by Harrell’s bootstrap optimism correction (1000 resamples, with the entire model-building procedure repeated within each resample) yielded an optimism-corrected C-index of 0.729, with CXCL8, IL-1A, and CCL2 remaining the three most frequently selected variables across replicates.

### 4.5. Copy Number Variation (CNV) Analysis

We estimated the initial CNV for each genomic region in individual cells using the inferCNV R package (v1.19.1) with the default parameters. We calculated the cumulative CNV for each cell. Briefly, the neutral copy number range was defined as the interval spanning twice the average standard deviation above and below the mean CNV of the reference group. The width of this neutral interval was defined as the single-copy change value, which served as the standard step unit for measuring CNV. Based on the defined step unit and neutral range, the inferred CNV (1.14.2) of each gene in individual cells was then categorized into six different CNV states: neutral (scored as 0, within the neutral range), single-copy loss (scored as 1, loss of one copy below the neutral range), complete loss (scored as 2, loss of two copies below the neutral range), single-copy gain (scored as 1, addition of one copy above the neutral range), two-copies gain two (scored as 2, addition of two copies above the neutral range), and high-level amplification (scored as 3, addition of more than two copies above the neutral range). Finally, the cumulative cellular CNV score was obtained by summing the weighted scores of the genes. This score serves as a quantitative indicator to assess the burden of genomic variation at the single-cell level.

### 4.6. Tumor Signature Scoring

After integrating the LAML dataset from The Cancer Genome Atlas and the normal whole blood dataset from the Genotype-Tissue Expression database (with random downsampling of Genotype-Tissue Expression samples to match the sample size of that of The Cancer Genome Atlas), we performed limma differential expression analysis to identify genes that were significantly upregulated in tumour tissues (log-fold change > 1, false discovery rate < 0.05). Subsequently, we employed the AddModuleScore function from the Seurat R package (v4.2.0) to calculate the tumour signature score for each cell based on the average expression level of the feature gene set, which was selected from the differential expression analysis within individual cells.

### 4.7. Tumor Cells Identification

To separate tumor cells from normal cells, we applied a co-embedding clustering-based transcriptional classification method, designated as “STR” (Single-cell Transcriptomic Reference-based classification). Using LSI, cells from each AML donors together with cells from the healthy sample were co-embedded on the reduced dimension space, respectively, followed by Seurat’s ‘RunUMAP’ with default parameters, performing Uniform Manifold Approximation and Projection (UMAP) as previously described. Disease-like cells of each AML sample were defined as cells in a cluster which contained more than 80% of cells from this AML sample. In contrast, healthy-like cells were cells that co-clustered in a same cluster with more than 80% of cells from the healthy donors. Cells that did not meet either threshold were classified as “Reference” cells.

### 4.8. Gene Set Variation Analysis

Gene set variation analysis (GSVA) was performed using the GSVA R package (v1.50.5) on the merged expression matrix of annotated T cells and neutrophils, with log-normalized expression values as input. The ssgsea algorithm was applied with a Gaussian kernel (kcdf = “Gaussian”) and default gene-set size filtering parameters (min.sz = 1, max.sz = Inf). Gene sets were obtained from the MSigDB database via the msigdbr R package (v10.0.1), comprising the C2:CP:KEGG_MEDICUS, C2:CP:KEGG_LEGACY, and C5:GO:BP collections. Pathway activity scores were computed on the combined expression matrix in a single GSVA run to ensure cross-cell-type comparability, and subsequently visualized separately for neutrophils and T cells.

### 4.9. Cell–Cell Communication Network Analysis

Cell–cell communication analysis. Intercellular communication networks were inferred using CellChat (v2.1.2) with the built-in human ligand–receptor database, CellChatDB.human. Cells were grouped by their annotated cell types, and communication probabilities between these populations were calculated based on the expression of ligands, receptors, and associated cofactors using the default triMean method implemented in CellChat. Only ligand-receptor interactions expressed in at least 10 cells (min.cells = 10) were considered to ensure robustness. Statistical significance of the inferred communications was determined using a permutation test (*n* = 100 permutations, *p* < 0.05). Signaling pathway-level communication networks were subsequently inferred and visualized following the standard CellChat workflow. To identify rewired networks, the healthy and AML datasets were merged to calculate differential communication probabilities, with *p* < 0.05 considered as significantly rewired.

### 4.10. Correlation Network Construction and Topological Analysis

To characterize global topological remodeling of the inflammatory microenvironment, undirected weighted correlation networks were constructed separately for the healthy control (*n* = 26) and AML (*n* = 46) groups based on the bone marrow supernatant concentrations of the same 20 inflammatory mediators quantified by multiplex Luminex profiling. Pairwise Spearman rank correlations were computed between all cytokine pairs based on their log_2_(x + 1)-transformed concentrations within each group, and an edge was drawn between two mediators when the correlation reached |r| ≥ 0.5 with *p* < 0.05; the absolute correlation coefficient served as the edge weight. Community structure (“modules”) was identified using the Louvain algorithm, and the degree of modular organization was quantified by the weighted network modularity Q, a standard network-science measure ranging from approximately −1 to 1, in which values near 0 indicate organization no greater than random expectation and values of approximately 0.2–0.4 denote pronounced community structure. All network analyses were performed in R using the igraph package (v1.5.0). Networks were visualized using a force-directed Fruchterman–Reingold layout, with edge width proportional to the absolute correlation coefficient and node color indicating community membership.

### 4.11. Statistical Analysis

All statistical analyses were performed using R software (v4.3.1). Continuous clinical variables are presented as medians (range) and compared using the Mann–Whitney U test or Kruskal–Wallis test, as appropriate. Correlations between continuous variables were assessed using Spearman analysis. Differential cytokine expression was adjusted using the Benjamini–Hochberg correction (FDR < 0.05). For survival analyses, cytokine concentrations were log_2_(x + 1)-transformed prior to univariate Cox proportional hazards regression and LASSO-penalized Cox regression, as detailed in Section 4.4. Prognostic performance was assessed by Kaplan–Meier analysis with the log-rank test, receiver operating characteristic (ROC) analysis, and Harrell’s C-index. Survival analyses included 45 evaluable patients (one excluded for no therapy and no follow-up). A two-sided *p* < 0.05 was considered statistically significant.

## 5. Conclusions

This study provides the first integrated single-cell and proteomic map of inflammatory network remodeling in AML bone marrow, revealing that immune dysregulation operates through three coordinated transformations rather than isolated cytokine perturbations: a cellular dominance shift from T-cells to myeloid populations, reconstruction of signaling networks into neutrophil-centric potential self-amplifying circuits, and acquisition of cell-type-dependent functional outputs. The inflammatory risk score derived from these network analyses captures prognostic information complementary to the ELN2022 genetic classification, particularly within the clinically ambiguous intermediate-risk category, where current systems offer limited guidance.

These findings establish inflammatory network pattern analysis as a novel dimension of AML biology with direct translational relevance. The identified self-amplifying axes, notably CXCL8–CXCR2 and CCL3–CCR1, represent rational candidates for immunomodulatory intervention aimed at dismantling pathogenic communication circuits rather than targeting individual cytokines. However, the present analyses are primarily correlative, and the IRS requires prospective multicenter protein-level validation before clinical implementation. Future studies incorporating functional validation of the proposed circuits, longitudinal profiling to track network evolution during treatment, and spatial transcriptomics to confirm physical proximity of interacting cells will be essential to translate these network-level insights into improved risk stratification and therapeutic strategies for AML patients.

## Data Availability

The raw data supporting the conclusions of this article will be made available by the authors on request.

## Supplementary Materials

The following supporting information can be downloaded at: https://www.mdpi.com/article/10.3390/ijms27198703/s1.

## Abbreviations

The following abbreviations are used in this manuscript:

AML	Acute myeloid leukemia
CXCL8	C-X-C motif ligand 8
CCL3	C-C motif ligand 3
CCL2	C-C motif ligand 2
IRS	Inflammatory risk score
CNV	Copy number variation
ROC	Receiver operating characteristic
