# Supplementary material for: Inflammatory Network Remodeling in AML Is Driven by Neutrophil-Centric Self-Amplifying Circuits
Source: Int J Mol Sci. 2026 Sep 29;27(19):8703. doi: 10.3390/ijms27198703 (PMC13665120; doi:10.3390/ijms27198703)
Supplement: Supplementary file 1 [file ijms-27-08703-s001.zip › ijms-4540508-supplementary.pdf]

Supplementary

1. Baseline characteristics of the 46 AML patients.

Table 1. Clinical Characteristics of the AML Patients at Baseline

(a) Baseline Characteristics of all patients (n=46)

Characteristic	All patients (N = 46)
Age, years, median (range)	51 (14–86)
Sex, n (%)	
Male	20 (43.5%)
Female	26 (56.5%)
WBC, $\times 10^9$/L, median (range)	12.1 (1.3–163.9)
Hemoglobin, g/L, median (range)	86.5 (32–142)
Platelets, $\times 10^9$/L, median (range)	36 (7–274)
Bone marrow blasts, %, median (range)	45.2 (15.5–97)
FAB subtype, n (%)	
M1	3 (6.5%)
M2	32 (69.6%)
M4	5 (10.9%)
M5	6 (13.0%)
ELN2022 risk category, n (%)	
Favorable	23 (50.0%)
Intermediate	13 (28.3%)
Adverse	10 (21.7%)
Molecular/cytogenetic findings, n (%)	
Tested	43/46
RUNX1::RUNX1T1	14 (30.4%)
NPM1	13 (28.3%)
CEBPA	7 (15.2%)
FLT3	9 (19.6%)
KIT	5 (10.9%)
IDH1/2	5 (10.9%)
CBFB::MYH11	2 (4.3%)
ASXL1	3 (6.5%)
DNMT3A	3 (6.5%)
TET2	3 (6.5%)
EZH2	2 (4.3%)
NRAS	2 (4.3%)
RUNX1	2 (4.3%)
Not performed	3 (6.5%)
Treatment, n (%)	
Intensive chemotherapy	36 (78.3%)
Hypomethylating agent + venetoclax	9 (19.6%)
Not documented	1 (2.2%)
Treatment response	
Evaluable	44/46
CR	33 (71.7%)
CRm	4 (8.7%)
CRi	1 (2.2%)
PR	3 (6.5%)
NR	3 (6.5%)
Not evaluable (NE)	2 (4.3%)
Survival status, n (%)	
Alive	33 (71.7%)
Deceased	12 (26.1%)
Lost to follow-up	1 (2.2%)

(b) Patients who underwent scRNA-seq (n = 3)

Patient	Age (years)	Sex	FAB	ELN202	Mutational profile	BM blasts (%)	OS (months)
P042	47	F	M4	Intermediate	NPM1; FLT3-ITD; DNMT3A	56.0	2.1
P044	29	M	M5	Intermediate	Not available	26.5	15.4
P043	29	F	M2	Favorable	CEBPA (bZIP)	78.0	1.4

Table S1. Clinical Characteristics of the AML Patients at Baseline.

(a) Baseline characteristics of all 46 newly diagnosed AML patients. Continuous variables are presented as median (range); categorical variables as n (%). Molecular/cytogenetic profiling was performed in 43 of 46 patients. All percentages (including mutation, treatment response, and survival status) are calculated

based on the total enrolled cohort (n = 46). The 1 lost-to-follow-up patient was excluded from the survival analysis (n = 45 evaluable).

(b) Individual clinical characteristics of the three patients (P042, P043, and P044) whose bone marrow samples underwent single-cell RNA sequencing; all three were part of the same 46-patient cohort summarized in panel (a). P044 declined clinical molecular/cytogenetic testing owing to financial constraints; the mutational profile is therefore not available.

Abbreviations: WBC, white blood cell count; Hb, hemoglobin; PLT, platelet count; BM, bone marrow; FAB, French-American-British; ELN, European LeukemiaNet; OS, overall survival; CR, complete remission; CRm, CR with incomplete count recovery; CRi, CR with incomplete hematologic recovery; PR, partial remission; NR, no response; NE, not evaluable.

2. Patient flowchart of the study cohort.

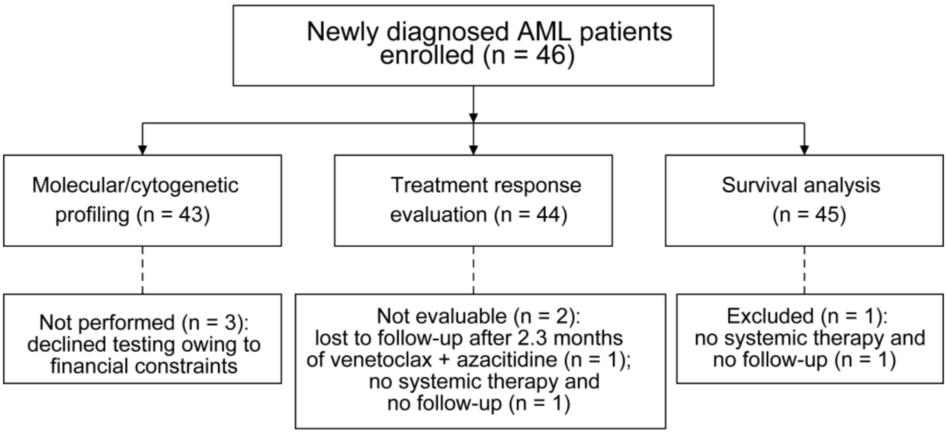

Figure S1. Patient flowchart of the study cohort. A total of 46 newly diagnosed AML patients were enrolled. Among them, 3 patients were excluded from molecular/cytogenetic profiling because they declined testing owing to financial constraints (n = 43 evaluable). For treatment response evaluation, 44 patients were evaluable, while 2 were not evaluable (1 patient was lost to follow-up after 2.3 months of venetoclax + azacitidine treatment, and 1 patient received no systemic therapy and was lost to follow-up). For survival analysis, 45 patients were included, and 1 patient was excluded due to receiving no systemic therapy and having no follow-up data. Abbreviations: AML, acute myeloid leukemia; NE, not evaluable.

3. **Patient flowchart of the study cohort.**

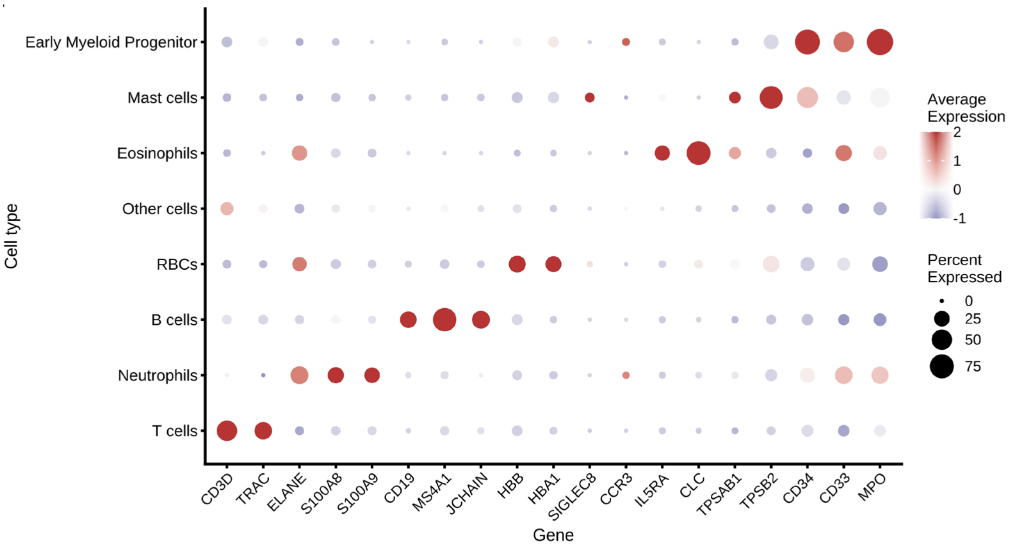

Figure S2. Marker-gene-based annotation of major hematopoietic populations.

Dot plot showing the average expression (color intensity) and percentage of expressing cells (dot size) of lineage-defining marker genes across the eight major hematopoietic populations identified in Figure 1A. T cells were annotated by CD3D and TRAC expression; neutrophils by ELANE, S100A8, and S100A9; B cells by CD19, MS4A1, and JCHAIN; RBCs (erythroid cells) by HBB and HBA1; eosinophils by SIGLEC8, CCR3, IL5RA, and CLC; mast cells by TPSAB1 and TPSB2; and early myeloid progenitors by CD34, CD33, and MPO. "Other cells" represent unclassified cells with no lineage-specific markers. Gene symbols are used for single-cell data. This panel supports the cell-type annotations used throughout the study.
